# Identification of LncRNAs and Functional Analysis of ceRNA Related to Fatty Acid Synthesis during Flax Seed Development

**DOI:** 10.3390/genes14050967

**Published:** 2023-04-24

**Authors:** Xinsen Yang, Caiyue Liu, Qiaoling Tang, Tianbao Zhang, Limin Wang, Lida Han, Jianping Zhang, Xinwu Pei

**Affiliations:** 1 Biotechnology Research Institute, Chinese Academy of Agricultural Sciences, Beijing 100081, China; 2 Crop Institute, Gansu Academy of Agricultural Sciences, Lanzhou 730070, China

**Keywords:** flax, lncRNA, fatty acids, ceRNA

## Abstract

Flax is a flowering plant cultivated for its oil and contains various unsaturated fatty acids. Linseed oil is known as the “deep-sea fish oil” of plants, and is beneficial to brain and blood lipids, among other positive effects. Long non-coding RNAs (lncRNAs) play an important role in plant growth and development. There are not many studies assessing how lncRNAs are related to the fatty acid synthesis of flax. The relative oil contents of the seeds of the variety Heiya NO.14 (for fiber) and the variety Macbeth (for oil) were determined at 5 day, 10 day, 20 day, and 30 day after flowering. We found that 10–20 day is an important period for ALA accumulation in the Macbeth variety. The strand-specific transcriptome data were analyzed at these four time points, and a series of lncRNAs related to flax seed development were screened. A competing endogenous RNA (ceRNA) network was constructed and the accuracy of the network was verified using qRT-PCR. MSTRG.20631.1 could act with miR156 on the same target, squamosa promoter-binding-like protein (SPL), to influence fatty acid biosynthesis through a gluconeogenesis-related pathway during flax seed development. This study provides a theoretical basis for future studies assessing the potential functions of lncRNAs during seed development.

## 1. Introduction

Flax is an important oil-producing and textile crop; its seeds are extracted for their oil and the stalks are stripped for fiber. Flax is typically classified as oil type, fiber type, or oil and fiber type based on its economic properties and uses [1]. The oil content of flax seeds is generally 40–50% and it is mainly composed of α-linolenic acid (ALA), palmitic acid (PAL), stearic acid (STE), oleic acid (OLE), and linoleic acid (LA) [2], with an average ALA content of 40–60%. ALA is an essential fatty acid that cannot be synthesized by the body, and its metabolites are known to lower blood lipids [3], reduce the risk of cardiovascular disease [4], fight inflammation [5], and prevent cancer [6]. This makes linseed oil a promising candidate for future use as a medicine and dietary supplement. Therefore, it is important to study the genes and regulatory pathways of fatty acid synthesis in flax.

LncRNAs have a length of more than 200 bp and are generally transcripts that cannot encode proteins [7]. They have a low expression and low sequence conservation. The in-depth study of lncRNAs has determined that lncRNAs affect gene expression through multiple pathways such as transcriptional interference [8] and transcription factor activation [9], while miRNAs interfere with mRNA function through various pathways such as target mRNA cleavage [10] and translation inhibition [11]. Additionally, lncRNAs and mRNAs act as response elements for competitive binding to microRNAs (miRNAs) in ceRNAs, reducing the ability of miRNAs to interfere with mRNAs and producing indirect regulatory effects [12]. LncRNAs are less commonly studied in plants than in animals, but a large number of lncRNAs have been reported in soybean [13], rice [14], rapeseed [15], maize [16], and peanuts [17]. However, there are no studies on lncRNAs related to seed development in flax.

Fatty acids play an essential role in plant growth and development, primarily as a major component of cell membranes and a provider of energy and signaling molecules [18]. Fatty acid biosynthesis is highly conserved across species and undergoes multiple catalytic reactions during elongation. The precursor of fatty acid synthesis is acetyl-CoA, which is first synthesized into malonyl-CoA by acetyl-CoA carboxylase, after which fatty acid synthase uses malonyl-CoA as a substrate for successive polymerization reactions to synthesize acyl chains at a rate of two carbons per cycle, leading to the synthesis of 16- to 18-carbon-saturated fatty acids [19]. The saturated fatty acids are then converted into monounsaturated fatty acids and polyunsaturated fatty acids by successive desaturation and extension reactions [20]. An increasing number of lncRNAs have been reported to be associated with fatty acid synthesis. Ma et al. constructed an interactive network involving 46 lncRNAs that may be involved in biological processes such as soybean fatty acid transport and lipid synthesis [13]. Tian et al. identified 35 lncRNAs regulating castor oil acid biosynthesis through transcriptome analysis [21]. Wu et al. identified 13 lncRNAs involved in the regulation of fatty acid biosynthesis through the co-expression network of Armeniaca sibirica kernels at five different developmental stages [22]. The genetic mechanism of fatty acid synthesis in flax has been well reported, with the identification of some key genes in the synthesis pathway [23,24,25]. However, there are few reports of lnRNAs associated with fatty acids in flax.

In this study, two flax varieties, Macbeth and Heiya NO.14, were used to analyze the relative oil content of the seeds at different developmental time points. Combined with strand-specific transcriptome data at different developmental periods, lncRNAs related to fatty acid synthesis during seed development were screened out. According to the ceRNA theory and a previous small RNA transcriptome data set, the ceRNA network was constructed. Through lncRNA, miRNA, and mRNA correlation analysis, the mechanism of lncRNA related to fatty acid synthesis was further analyzed. The goal is to reveal the relationship between fatty acids and lncRNAs in flax seed development, analyze the mechanism behind how lncRNAs regulate flax fatty acids, and provide a theoretical basis for improving flax fatty acids.

## 2. Materials and Methods

### 2.1. Sample Collection and Seed Oil Content Determination

In order to clarify the regulation mechanism of flax fatty acid synthesis, oil-flax variety Macbeth (M) and fiber-flax variety Heiya NO.14 (H) were used as study materials. The two varieties were selected because Macbeth has a high oil content (467 g/kg), high linolenic acid content (56.8%), large seed size (6.0 g/1000 seed weight), lodging resistance, disease resistance, and high yield, and it is suitable for a variety of soils; it has been widely cultivated since its development in Canada in 2002 [26]. Heiya 14, developed by China, has a strong stress resistance, wide adaptability, high rate of long hemp (18.4%), and high fiber strength (26.4 kg), and is part of a high quality, high resistance, and high yield variety [27]. The varieties were planted in the field trial station of the Chinese Academy of Agricultural Sciences in Langfang City, Hebei Province, China (39°35′28″ N, 116°35′53″ E). The total RNA was extracted from the fruit at 5 day (M5, H5), 10 day (M10, H10), 20 day (M20, H20), and 30 day (M30, H30) after flowering, and strand-specific sequencing was performed. The capsule seeds of the four periods were taken out and dried, and the relative oil content was determined using an infrared analyzer DA7200 (Perten Instruments Co., Ltd., Stockholm, Sweden). At each set time point, the analysis was repeated four times and the mean values were used as the final content.

### 2.2. RNA Extraction, Quality Control, and Sequencing

The total RNA was extracted using a plant tissue RNA extraction kit (DP419, Beijing Tiangen Company, Beijing, China). RNA degradation and contamination were detected on 1.5% agarose gel. The RNA concentration and purity were measured using a NanoDrop 2000 Spectrophotometer (Thermo Fisher Scientific, Wilmington, DE, USA), and the RNA integrity was assessed using the RNA Nano 6000 Assay Kit of the Agilent Bioanalyzer 2100 System (Agilent Technologies, Santa Clara, CA, USA). After passing a quality control screening, 1.5 μg of RNA was used for each sample for library construction. rRNA removal was performed using a Ribo-Zero rRNA removal kit (Epiccentre, Madison, WI, USA). Sequencing libraries were generated using the nebnexr Ultra^TM^ Directed RNA Library Prep Kit for IlluminaR (NEB, Ipswich, MA, USA), according to the manufacturer’s recommendations. A total of 16 libraries were constructed for the two varieties at four time points, and index codes were added to each sample in the attribute sequence. The samples at each time point were taken from a fruit mixture of three plants of the same species, with two fruits taken from each plant. To preferentially select inserts of 150–200 bp in length, library fragments were purified with AMPure XP Beads (Beckman Coulter, Beverly, MA, USA). PCR was then performed with 3 μL of USER enzyme (NEB) incubated with the size-selected, adapter-ligated cDNA for 15 min at 37 °C. PCR was then performed with Phusion high-fidelity DNA polymerase, universal PCR primers, and index (X) primers. The PCR products were finally purified using the AMPure XP system, and the library quality was assessed using an Agilent Bioanalyzer 2100 and qPCR. Clustering of the index-encoded samples was performed in the acBot Cluster Generation System using the TruSeq PE Cluster Kitv3-cBot-HS (Illumina, San Diego, CA, USA), according to the manufacturer’s instructions. After cluster generation, library preparations were sequenced on the Illumina Hiseq platform (Biomarker Technologies, Beijing, China) in order to generate paired-end reads.

### 2.3. Strand-Specific Transcriptome Data Analysis

The raw reads of the strand-specific transcriptome were removed from the raw reads containing adapters and those of a low quality (the ratio of N was greater than 10%, and the number of bases with Q ≤ 10 accounted for more than 50% of the entire read), after which the clean read was obtained. *Linum usitatissimum* (https://genome.jgi.doe.gov/portal/pages/dynamicOrganismDownload.jsf?organism=PhytozomeV11 (accessed on 24 January 2023)) was used as the reference genome, and TopHat2 [28] was used to align the clean reads with the reference genome sequence. The ones that could be compared were mapped reads. Cufflinks were used to concatenate and annotate the mapped reads. In the basic screening of lncRNAs, transcripts with a length of ≥200 bp and the number of exons ≥2 were selected. Cuffdiff [29] was used to calculate FPKM as an indicator to measure the level of transcripts or gene expression. Transcripts with FPKM ≥ 0.1 were screened. Transcripts that could not be coded were screened for newly predicted lncRNAs using CPC, CNCI, CPAT, and PFMA protein domain analysis [30,31,32,33]. The lncRNA annotation file is shown in the Supplementary File S1.

### 2.4. LncRNA Differential Expression Analysis and Functional Annotation

During the detection of differentially expressed lncRNAs, DESeq was used for differential analysis [34], and we used fold change (FC) ≥ 2 and FDR < 0.05 as indicators for differential screening. Cis-acting is a mode of action of lncRNAs on target genes, which means that lncRNAs act on one or more genes transcribed at the same site [35]. We predicted the upstream and downstream 100 kb range of lncRNAs using perl script Neighboring genes, which were used as the lncRNA cis target genes. The target genes were aligned with NR [36], GO [37], and KEGG [38] database sequences using BLAST [39] to obtain annotated information about the lncRNA target genes.

### 2.5. Construction of ceRNA Network Related to Seed Development

From the above analysis results, we found that the amount of differential lncRNA data was most abundant at M5 and M30. To further analyze how lncRNAs are related to fatty acid accumulation during seed development in the Macbeth variety, we selected the strand-specific transcriptome and small RNA transcriptome data of Macbeth with a high oil content at 5 day and 30 day for lncRNA-miRNA-mRNA association analysis and constructed ceRNA networks, in which small RNA and mRNA datasets were obtained from the previous studies in our lab [40,41]. The lncRNA was re-annotated and the annotation information is shown in Supplementary File S2. We reset the screening criteria for differential lncRNAs to fold change ≥ 2 and FDR < 0.01, and used |log2(FC)| ≥ 1.00 and FDR ≤ 0.01 for differential miRNAs. TargetFinder [42] was used to predict the miRNA−lncRNA and miRNA−mRNA relationship pairs, and the perl script was used to predict lncRNA−mRNA relationship pairs. The number of identical miRNAs between ceRNAs was more than 5, the hypergeometric test *p*-value < 0.01, and the corrected FDR value < 0.01. The ceRNA network was visualized using Cytoscape 3.8.2 software.

### 2.6. Validation of the ceRNAs Network by qRT-PCR

The relative expression of lncRNAs, miRNAs, and mRNAs during seed development was assessed by quantitative real-time polymerase chain reaction (qRT-PCR). The total RNA of lncRNAs and mRNAs was extracted using the RNA Easy Fast Plant Tissue Kit (DP419, Beijing Tiangen, Beijing, China) and the total RNA of the miRNAs was extracted using the UNlQ-10 column-based Trizol Total RNA Extraction Kit (B511321, Bioengineering Shanghai Co., Ltd., Shanghai, China). The stem-loop reverse transcription primers for miRNAs were designed using Primer Premier 5.0. SnapGene software was used to design the primers for the lncRNAs and mRNAs. Quantitative analysis was performed using ABI Quant Studio 6 fluorescent quantitative PCR (Applied Biosystems by Life Technologies, Carlsbad, CA, USA). The relative RNA expression levels were calculated using the 2^−ΔΔCT^ method [43]. Three replicates were set up for each data set and the standard deviations were calculated. Actin is an internal reference gene for lncRNAs, miRNAs, and mRNAs. The primer sequences are included in Supplementary File S3.

## 3. Results

### 3.1. Oil Content of Macbeth and Heiya NO.14 Varieties at Different Development Stages

**Figure 1:**
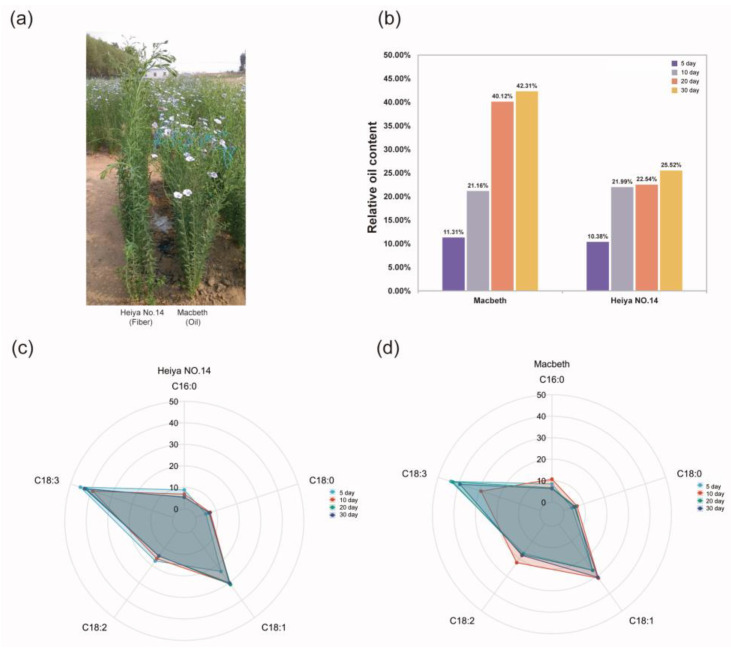
The difference between Macbeth and Heiya NO.14 regarding the relative oil content and fatty acid content. (**a**) Field phenotypes of Macbeth and Heiya NO.14, with the fiber variety Heiya No.14 on the left and the oil variety Macbeth on the right. (**b**) Relative oil content of Macbeth and Heiya NO.14 at 5 day, 10 day, 20 day, and 30 day. (**c**,**d**) The relative content of fatty acid in Heiya NO.14 and Macbeth at 5 day, 10 day, 20 day, and 30 day, respectively.

The Macbeth (M) and Heiya NO.14 (H) varieties were used in our study (Figure 1a). As the seed developed, the seeds became fuller and the oil content gradually increased. The relative oil content of the Macbeth and Heiya NO.14 varieties was only 11.31% and 10.38%, respectively, at 5 day after flowering. By 30 day after flowering, the relative oil content of the Macbeth variety was 42.31%, while that of the Heiya NO.14 variety was only 25.52% (Figure 1b). The relative oil content of Macbeth increased continuously from 5 to 20 day, but did not change significantly from 20 to 30 day. The relative oil content of Heiya NO.14 did not significantly change after 10 day. The analysis of fatty acid types in different development periods indicated that the percentages of different types of fatty acids in Heiya NO.14 were the same at each stage, and there was no significant change (Figure 1c). In the Macbeth variety, the relative content of ALA decreased by approximately 10% at 10 day. During this process, the proportions of PAL, STE, OLE, and LIO all increased, while at 20 day, the relative percentage of ALA increased to approximately 40% (Figure 1d).

### 3.2. Analysis of the Strand-Specific Transcriptome Data

**Figure 2:**
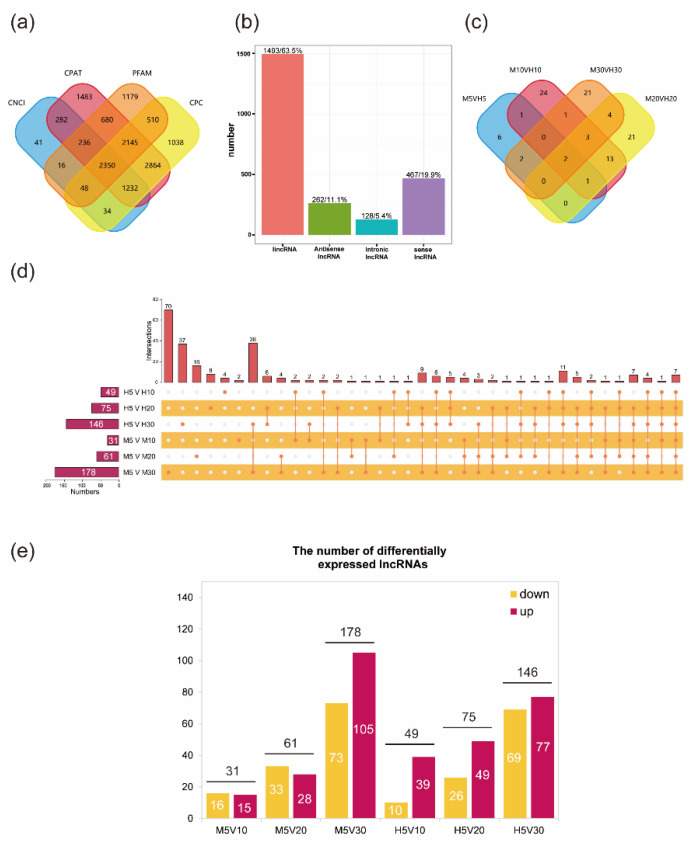
Strand-specific transcriptome data analysis. (**a**) Venn diagram drawn by the results of CPC, CNCI, CPAT, and PFAM. (**b**) lncRNA category diagram. (**c**) Venn diagram of the number of differential lncRNAs between Macbeth and Heiya NO.14 at 5 day, 10 day, 20 day, and 30 day. (**d**) Upset diagram of differentially expressed lncRNAs between Macbeth and Heiya NO.14 in four stages. The 5 day data of Macbeth and Heiya NO.14 were used as a control, and were compared with the data of 10 day, 20 day, and 30 day, respectively. (**e**) The number of differentially expressed lncRNAs.

The raw data of the 16 strand-specific libraries were quality-controlled to obtain 188.35 GB clean reads, with more than 81,000,000 clean reads for each sample, all with a GC content greater than 47% and Q30 above 88% (Supplementary File S4), which indicates that the clean reads were of a high quality. The clean reads from each sample were sequenced against the developed reference genome separately and the matching efficiency ranged from 74.14% to 85.91%. After basic screening of the transcripts, we used CNCI, CPC, PFAM, and CPAT to screen for their coding ability, and obtained 4236, 7344, 11,272, and 10,218 lncRNAs, respectively. We selected the intersection of the four methods as the newly predicted lncRNA for the subsequent analysis, and a total of 2350 new predicted lncRNAs were obtained (Supplementary File S5) (Figure 2a). Of these, the number of intergenic lncRNAs (lincRNAs) was at most 1493 (63.5%), while 262 (11.1%) belonged to antisense-lncRNAs, 128 (5.4%) belonged to intronic-lncRNAs, and 467 (19.9%) belonged to sense lncRNAs (Figure 2b). The maximum number of lncRNA exons was 12, and 54.47% of the lncRNA exons had only 2 exons. In addition, 90.16% of lncRNA open reading frames were less than 125 bp in length. Most lncRNAs were 700–900 bp in length, with a median length of 875 bp. We compared the basic characteristics of the two varieties and found that there was no significant difference in the lncRNAs screened between the two varieties (Supplementary File S6). Among the newly predicted lncRNAs, 2152 lncRNAs predicted 23,339 cis-target genes (Supplementary File S7).

### 3.3. Differential lncRNA Expression Analysis during Seed Development between the Macbeth and Heiya NO.14 Varieties

To more clearly compare the differences between the Macbeth and Heiya NO.14 varieties at each time point, data from 5 day, 10 day, 20 day, and 30 day after flowering were compared, using Macbeth as a control. Two lncRNAs (TCONS_00107244 and TCONS_00245620) were found to be significantly down-regulated at all four stages. We also found the highest number of specifically expressed lncRNAs at 10 day with 24, and the least at 5 day with 6, and 21 at both 20 day and 30 day (Figure 2c).

To investigate the differences between Macbeth and Heiya NO.14 during seed development, we used the 5 day data from both varieties as a control and compared them with the data from 10 day, 20 day, and 30 day (Figure 2d,e). We found that at 10 day, Macbeth had 31 differentially expressed lncRNAs (15 up-regulated and 16 down-regulated), which was less than the 49 (27 up-regulated, 18 down-regulated) in Heiya NO.14. In Macbeth, the highest number of 30 day significantly differential lncRNAs was 178 (105 up-regulated and 73 down-regulated), of which 70 were specifically differentially expressed. In Heiya NO.14, the number of significantly differentially expressed lncRNAs was also highest at 30 day, with 146 (77 up-regulated and 69 down-regulated) and 37 specifically differentially expressed, which was lower than in Macbeth. The number of differentially expressed lncRNAs gradually increased over time, with an approximate 4.74-fold increase from M10 to M30 compared with 1.98-fold for Heiya NO.14. This indicates that Macbeth invoked more lncRNAs involved in seed development than Heiya NO.14 from 5 day to 30 day after flowering. We screened for two down-regulated lncRNAs (TCONS_00180560 and TCONS_00213922) in Macbeth and Heiya NO.14 that were differentially expressed in all three stages. Based on the trend of seed oil content growth, we screened two shared lncRNAs (TCONS_00021781 and TCONS_00134305) in Macbeth and Heiya NO.14 at 5 day and 10 day, both of which were significantly up-regulated and whose fragments per kilobase of transcript per million fragments mapped (FPKM) peaked at 10 day (Supplementary File S8).

**Figure 3:**
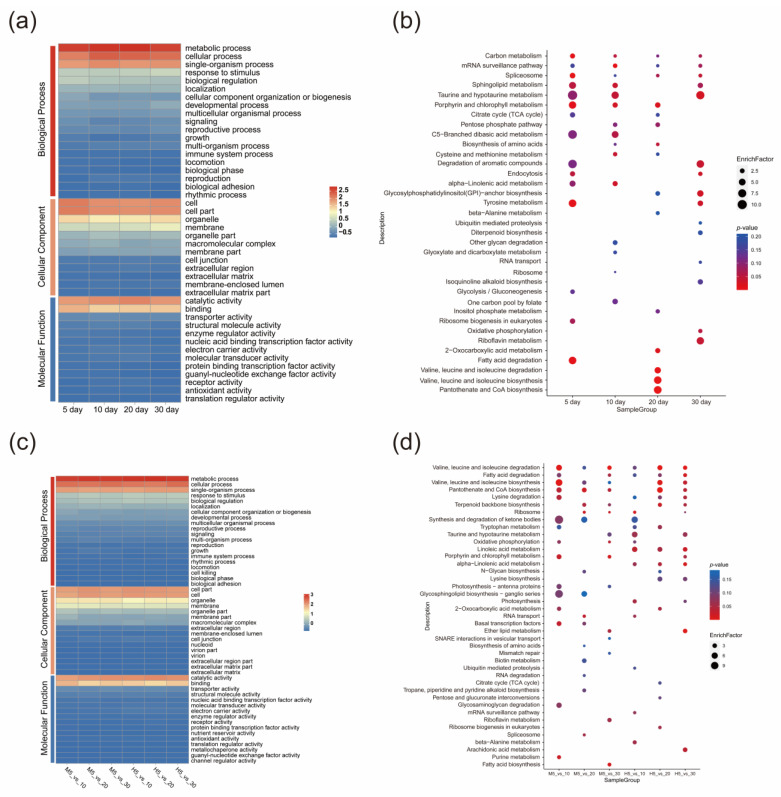
Functional analysis of lncRNAs. (**a**,**b**) GO and KEGG functional annotations of cis-target genes at four time points with Macbeth as the control, respectively. Heatmaps are drawn with cis-target gene numbers in a, and target gene numbers are normalized according to the columns. (**c**,**d**) GO and KEGG annotations of cis-target genes in Macbeth and Heiya NO.14 compared with respective 5 day data as the controls and at 10 day, 20 day, and 30 day. The number of target genes is normalized according to the column.

To explore the functional differences of lncRNAs between the two cultivars at four time points, we performed functional annotation of the target genes of differential lncRNAs based on the GO database with Macbeth as the control, and the results showed that the cis-target genes of the two cultivars were annotated in 44 terms and the number of terms annotated at each time point was approximately the same. We showed that the GO results were mainly enriched in terms of metabolic process, cellular process, single-organism process, cell, cell part, organelle, membrane, catalytic activity, and binding, etc. (Figure 3a). The KEGG results showed that more pathways were annotated at 10 day. We selected the 15 most significant pathways at each time point for further analysis, and found that α-linolenic acid metabolism was more enriched at 5 day and 10 day than at 20 day and 30 day (Figure 3b). This could be related to the decrease in the relative content of linolenic acid in M5 to M10.

We compared the 5 day data of Macbeth and Heiya NO.14 with the 10 day, 20 day, and 30 day data. The results of GO showed that there was no significant difference in terms of the genes enriched at different developmental stages between the two cultivars. The metabolic process, cellular process, and single-organism process, as well as the cell part, cell, and organelle had the most genes, and the number of genes involved in catalytic activity, binding and transporter activity was the highest in terms of molecular function. (Figure 3c). KEGG functional annotation showed that linoleic acid metabolism and α-linolenic acid metabolism were significantly enriched during all stages of Heiya NO.14 development, but not in Macbeth (Figure 3d) (Supplementary File S9). These results suggest that the unsaturated fatty acids in Heiya NO.14 are involved in other life activities through metabolic reactions, whereas Macbeth may retain these unsaturated fatty acids.

### 3.4. Construction and Validation of ceRNA Network Related to Seed Development in Macbeth

**Figure 4:**
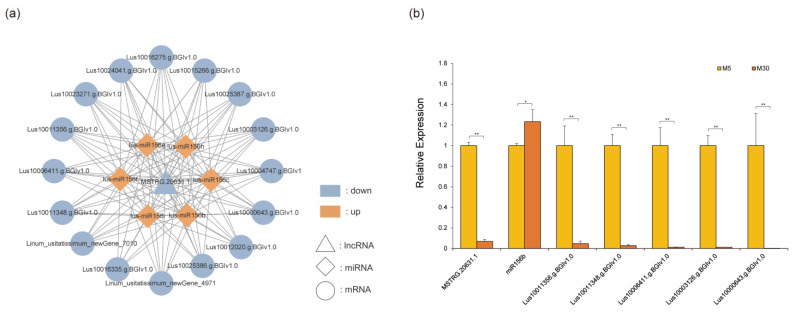
ceRNA network related to seed development in Macbeth. (**a**) ceRNA network. Triangles represent lncRNAs, diamonds represent miRNAs, and circles represent mRNAs. Blue indicates down-regulation and orange indicates up-regulation. (**b**) ceRNA network validation by qPCR. Yellow represents the fruit of Macbeth at 5 day and orange represents the fruit of Macbeth at 30 day. On the X-axis are the different RNAs, and on the Y-axis are the relative expression levels calculated using the 2^−ΔΔCT^ method. A star on top of each bar indicates statistically significant difference (*p* < 0.05). Two stars indicate extremely significant difference (*p* < 0.01).

Interactions between lncRNAs and miRNAs play an important role in regulating plant growth and development and in responding to stress [44]. To construct the ceRNA network, we re-annotated the lncRNA of the strand-specific sequencing (Supplementary File S10). Based on ceRNA theory, we found a regulatory relationship between 8 lncRNAs, 22 miRNAs, and 65 mRNAs differentially expressed in the Macbeth variety at 5 day and 30 day (Supplementary File S11). We obtained a ceRNA network consisting of 1 lncRNA, 6 miRNAs, and 15 mRNAs through negative regulatory screening (Figure 4a). In our network, six miRNAs (lus-miR156b, lus-miR156c, lus-miR156e, lus-miR156f, lus-miR156h, and lus-miR156i) were classified as being part of the miR156 family through sequence analysis, and their maturation sequences were consistent. Lus10006411.g.BGIv1.0 and Lus10011356.g.BGIv1.0 were annotated as SPL2, Lus10000643.g.BGIv1.0 was annotated as SPL6, and Lus10003126.g.BGIv1.0 and Lus10011348.g. BGIv1.0 were both annotated as SPL16. We used the seed of Macbeth at 5 day and 30 day after flowering and selected MSTRG.20631.1, miR156, and five SPL genes in the ceRNA network to verify the ceRNA network using qRT-PCR (Figure 4b). The results demonstrate that MSTRG.20631.1 was significantly down-regulated, miR156 was significantly up-regulated, and five SPL genes were significantly down-regulated. This is consistent with what we found.

## 4. Discussion

Flax seed oil is rich in various unsaturated fatty acids, especially ALA [45]. To investigate the role of lncRNA in flax seed development and fatty acid synthesis, we determined the relative oil content of the oil-flax variety Macbeth and the fiber-flax variety Heiya NO.14 at different development stages. The function of lncRNAs in these two varieties was analyzed using a strand-specific transcriptome technique. We found that the relative oil content of both varieties increased by approximately 10% between 5 day and 10 day. The Macbeth variety maintained this trend from 10 to 20 day, reaching 40.11% by 20 day and had no major changes between 20 and 30 day, while Heiya NO.14 did not experience a major increase in relative seed oil content after 10 day of flowering. This demonstrates that Macbeth experienced a rapid increase in the expression of fatty acid synthesis-related genes from 5–20 day after flowering, while Heiya NO.14 experienced this from 5–10 day. The results of the analysis of the fatty acid components of the two varieties at each time point found that the relative content of various fatty acids in Heiya NO.14 did not change much, indicating that the accumulation of various fatty acids in Heiya NO.14 occurred synchronously during the entire fatty acid accumulation process. In the Macbeth variety, the relative ALA content decreased by approximately 10% at 10 day, while the relative ALA content increased to approximately 40% at 20 day. Therefore, we speculate that 10–20 day is an important time period for ALA accumulation in the Macbeth variety, which is consistent with previous research [46].

We analyzed the basic characteristics of lncRNAs in flax, in which lincRNAs were found to be the most abundant, which is consistent with previous findings in soybean [47], peanut [48], and wheat [49]. The median lncRNA length is longer in flax than in rice [50], which could be due to differences between species. We compared the lncRNAs in the two varieties at three development periods and found that the FPKM of TCONS_00107244 was almost 0 in Heiya NO.14, while in Macbeth, TCONS_00107244 had a slow upward trend from 5 day to 20 day, and decreased at 30 day. This is similar to the increasing trend of oil content observed in the Macbeth variety. The target genes of TCONS_00107244 were annotated in terms of the chlorophyll cycle (GO:0033354), integral membrane composition (GO:0016021), endoplasmic reticulum membrane (GO:0005789), and plasma membrane (GO:0005886). The transport of lipids primarily occurs in the chloroplast and the back of the endoplasmic reticulum [51], and most of the membrane lipids in the chloroplast were assembled on the membrane [52]. Therefore, TCONS_00107244 could play a role in the synthesis of membrane lipids in the Macbeth variety. Combined with the above analysis, TCONS_00107244 could affect the accumulation of fatty acid content in seeds in multiple pathways, such as membrane lipid synthesis, chlorophyll transformation, and material transport, resulting in a higher oil content in Macbeth than Heiya NO.14.

Acetyl COA produced by glucose metabolism is an important substrate in fatty acid synthesis [53]. α-glucan, water dikinase (GWD) is a starch-degrading enzyme that transfers the β-phosphate of phosphohistidine to the C6 position of the glucose group and phosphorylates it [54,55]. β-Glucosidase acts on aryl-β-glucosides and cellobiose to release glucose [56,57]. We found that the cis-target genes of TCONS_00180560 and TCONS_00213922 act on GWD and β-glucosidase, respectively, and their expression levels gradually decreased during the development of the two varieties. Relative to 5 day, TCONS_00180560 was significantly down-regulated (approximately 27-fold) at M10 and was no longer expressed at M20 and M30. TCONS_00213922 was no longer expressed in Macbeth at 30 day and had a lower FPKM at 10 and 20 day. In Heiya NO.14, TCONS_00180560 and TCONS_00213922 were barely expressed in H10 and were not expressed in H20 and H30. This suggests that Macbeth relies on TCONS_00180560 and TCONS_00213922 to accumulate more glucose during the degradation of starch and aryl-β-glucosides via GWD and β-glucosidase. This could have contributed to the accumulation of fatty acids in the seeds of the Macbeth and Heiya NO.14 varieties at 5 day.

We found that the relative oil content of both varieties increased from 5–10 day, and we screened two lncRNAs (TCONS_00021781 and TCONS_00134305) that they shared during this process. Functional analysis found that the target genes of TCONS_00021781 were annotated to entries related to chlorophyllase (EC: 3.1.1.14). Chlorophyllase catalyzes the degradation of chlorophyll to form chlorophyll esters, during which a large amount of phytol is released to participate in fatty acid phytate biosynthesis [58]. Therefore, TCONS_00021781 could be associated with a sharp increase in relative oil content in seeds from 5–10 day. The target gene of TCONS_00134305 was annotated in the cell membrane (GO:0016020)-related entry, which is associated with fatty acid ω-hydroxylase. Fatty acid ω-hydroxylase can use fatty acid as a substrate to participate in the synthesis of suberin and cutin of the seed coat [59], which could be negatively correlated with fatty acid accumulation.

LncRNAs have been reported to function as ceRNAs in the post-transcriptional regulation of miRNA-mediated gene expression. Here, for the first time, we constructed a ceRNA network based on strand-specific transcriptome data and small RNA data. The miR156-SPL regulatory module is involved in multiple processes of plant growth and development [40,60]. The expression levels of some SPLs in early seed development were higher than in later stages [61], and this expression pattern could be regulated by miR156 [62]. In our network, Lus10003126.g.BGIv1.0 (SPL16), Lus10006411.g. BGIv1.0 (SPL2), Lus10000643.g. BGIv1.0 (SPL6), Lus10011348.g. BGIv1.0 (SPL16), and Lus10011356.g.BGIv1.0 (SPL2) were significantly down-regulated from 5 to 30 day after flowering in Macbeth. miR156-SPL is a central module that affects multiple plant traits [63] and regulates plant salt tolerance [64], germination [65], and transition from vegetative to reproductive growth through SPL [66]. This regulation could be triggered by the transition between sucrose and glucose [67]. As a key signaling molecule and carbon source for most plant cells, sugar is closely related to lipid synthesis [68]. Our previous study found that the overexpression of miR156 could reduce the oil content of Arabidopsis thaliana by regulating SPL [40]. In this study, using bioinformatics analysis, we found that MSTRG.20631.1 and SPL competed to bind miR156. Functional annotation revealed that MSTRG.20631.1 target genes were involved in glycolysis (GO: 0006096), transport (GO: 0006810), and other pathways. This indicates that MSTRG.20631.1 could act on SPL by regulating the pathways related to sugar metabolism in plants, thereby regulating growth and development. During fatty acid accumulation in flax seeds, MSTRG.20631.1 positively regulates SPL to inhibit fatty acid accumulation. Plants significantly up-regulated the expression of miR156 due to growth and development. The combination of MSTRG.20631.1 and miR156 reduced the enhancement effect of MSTRG.20631.1 on SPL, and the highly expressed miR156 also significantly enhanced the inhibitory effect of SPL, which positively affects fatty acid accumulation. However, the specific regulatory mechanism needs to be further verified.

## 5. Conclusions

We analyzed the relative oil content of the seeds of the oil flax variety Macbeth and the fiber variety Heiya NO.14 at 5 day, 10 day, 20 day, and 30 day. It was found that the high expression of fatty acid accumulation-related lncRNAs in the Macbeth variety primarily occurred from 5–20 day, while Heiya NO.14 primarily occurred from 5–10 day. In Macbeth, ALA synthesis-related genes were highly expressed at 10–20 day. We performed strand-specific transcriptome analysis of two flax varieties based on the relative oil content trend. Based on the functional annotation information of GO, KEGG, and NR, a group of lncRNAs that may be related to fatty acid accumulation were screened. To further understand lncRNA function, we constructed a ceRNA network and found that MSTRG.20631.1 and miR156 co-regulated SPL6, SPL16, and SPL2, which play an important role in fatty acid accumulation by regulating the glucose metabolism-related pathways during flax seed development. We also validated our network by qRT-PCR. Our study provides a theoretical basis for studying the role of lncRNAs in fatty acid biosynthesis.

## Data Availability

The Strand-specific transcriptome raw data of Heiya NO.14 obtained at different developmental stages are available in the NCBI database (https://www.ncbi.nlm.nih.gov/geo/query/acc.cgi?acc=GSE130378 (accessed on 24 January 2023)). The Strand-specific transcriptome and small RNA raw data of Macbeth have been deposited into SRA database (https://www.ncbi.nlm.nih.gov/sra (accessed on 24 January 2023)), accession number in Supplementary File S12.

## Supplementary Materials

The following supporting information can be downloaded at: https://www.mdpi.com/article/10.3390/genes14050967/s1. Supplementary File S1: Annotation of LncRNA; Supplementary File S2: Annotation of LncRNA in ceRNA; Supplementary File S3: Primers used in the article; Supplementary File S4: Quality control table of strand-specific sequence; Supplementary File S5: Sequence of LncRNA; Supplementary File S6: Basic characteristics of LncRNA in Macbeth and Heiya NO.14; Supplementary File S7: Cis-target gene of LncRNA; Supplementary File S8: The FPKM of LncRNA at different time periods in the two varieties; Supplementary File S9: Functional annotation of Cis-target gene; Supplementary File S10: Sequence of LncRNA in ceRNA; Supplementary File S11: RNA of ceRNA network; Supplementary File S12: Accession number of Macbeth‘ strand-specific transcriptome data in SRA database.
